# Djulis (*Chenopodium formosanum*) Extract as a Promising Natural Agent Against Skin Aging

**DOI:** 10.3390/molecules30153209

**Published:** 2025-07-31

**Authors:** Jia-Ling Lyu, Po-Yuan Wu, Hsiao-Fang Liao, Chia-Lin Lee, Kuo-Ching Wen, Chang-Cheng Chang, Hsiu-Mei Chiang

**Affiliations:** 1 Department of Cosmeceutics, China Medical University, Taichung 406, Taiwanu111307801@cmu.edu.tw (H.-F.L.); chlilee@mail.cmu.edu.tw (C.-L.L.); kcwen0520@mail.cmu.edu.tw (K.-C.W.); changcc1975@gmail.com (C.-C.C.); 2 School of Medicine, China Medical University, Taichung 404, Taiwan; wu.poyuan@gmail.com; 3 Department of Dermatology, China Medical University Hospital, Taichung 404, Taiwan; 4 Ph.D. Program for Biotechnology Industry, China Medical University, Taichung 406, Taiwan; 5 Chinese Medicine Research and Development Center, China Medical University Hospital, Taichung 404, Taiwan; 6 Chinese Medicine Research Center, China Medical University, Taichung 404, Taiwan; 7 Aesthetic Medical Center, China Medical University Hospital, Taichung 404, Taiwan

**Keywords:** *Chenopodium formosanum*, photoaging, phytochemicals, antioxidant, anti-inflammatory, matrix metalloproteinases, advanced glycation end products

## Abstract

Photoaging, predominantly induced by ultraviolet radiation, is a primary driver of premature skin aging, characterized by complex molecular mechanisms including oxidative stress, inflammation, matrix metalloproteinase activation, and extracellular matrix degradation. Consequently, there is growing scientific interest in identifying effective natural agents to counteract skin aging and photoaging. Djulis (*Chenopodium formosanum*), an indigenous Taiwanese pseudocereal from the Amaranthaceae family, has emerged as a promising candidate for skincare applications because of its rich phytochemicals and diverse bioactivities. This review describes the current understanding of the molecular mechanisms underlying photoaging and examines the therapeutic potential of djulis extract as a multifunctional agent for skin aging. Its mechanisms of action include enhancing antioxidant defenses, modulating inflammatory pathways, preserving the extracellular matrix, and inhibiting the formation of advanced glycation end products. Bioactive constituents of djulis extract, including phenolic compounds, flavonoids, and betanin, are known to exhibit potent antioxidant and photoprotective activities by modulating multiple molecular pathways essential for skin protection. The bioactivities of djulis in in vitro and animal studies, and four skin clinical trials of djulis extract products are presented in this review article. Ultimately, this review provides an overview that supports the potential of djulis extract in the development of evidence-based skincare formulations for the prevention and treatment of skin aging.

## 1. Introduction

Aging is a biological process characterized by the progressive accumulation of cellular damage over time, leading to dysfunction at the cellular, tissue, and organ levels, which increases susceptibility to disease and mortality. Cutaneous aging is a complex biological process driven by both intrinsic (chronological) factors and extrinsic environmental stressors, which collectively contribute to structural and morphological alterations within the skin matrix. Among these, solar ultraviolet (UV) radiation is the predominant extrinsic aging, known as photoaging [1]. These processes significantly impact both the skin’s aesthetic appearance and its fundamental physiological functions.

Excessive UV exposure stimulates the overproduction of reactive oxygen species (ROS) in skin cells, which in turn can lead to hyperpigmentation, skin inflammation, immunosuppression, accelerated photoaging, and carcinogenesis [2]. The clinical manifestations of photoaging include the formation of wrinkles, solar lentigines, telangiectasias, and diminished elasticity [3]. Functionally, photoaged skin exhibits an impaired barrier, immunological dysfunction, and reduced capacity for vitamin D synthesis. Furthermore, photodamaged skin demonstrates impaired wound healing responses, deficient angiogenesis, and increased susceptibility to external irritants and various dermatological pathologies [1,4,5].

In response to these challenges, phytochemical-rich botanical extracts have shown considerable therapeutic efficacy in addressing various cutaneous disorders [6,7,8]. Among these, djulis (*Chenopodium formosanum*), an indigenous Taiwanese pseudocereal, is recognized for containing bioactive compounds with significant potential for dermatological applications [9,10]. This review aimed to critically examine the current scientific literature supporting djulis extract as an anti-skinaging and anti-photoaging therapeutic agent, elucidating its bioactive constituents, underlying molecular mechanisms of action, and therapeutic potential in photoaging intervention.

## 2. Mechanisms of Photoaging

### 2.1. Ultraviolet Radiation

Solar ultraviolet radiation is categorized into three spectral regions based on its biological effects: UVC (200–280 nm), UVB (280–320 nm), and UVA (320–400 nm). UVB radiation primarily affects the epidermal layer, where it induces direct DNA damage and erythematous responses, whereas UVA radiation penetrates more deeply into the dermal compartment, contributing significantly to photoaging processes and collagen degradation [10,11,12]. Chronic exposure to UVA can generate excessive oxidative stress, initiating a cascade of reactions that indirectly cause oxidation of intracellular lipids, proteins, and nucleic acids [13]. The substantial production of ROS by UVA irradiation can also trigger the intrinsic apoptotic pathway by altering mitochondrial membrane permeability, leading to cytochrome C efflux and the activation of downstream caspases [1]. Conversely, UVB radiation is readily absorbed by DNA bases, proteins, and aromatic amino acids, leading to direct photochemical damage and the formation of DNA photoproducts [10,14,15]. In contrast, UVA radiation primarily interacts with endogenous chromophores such as porphyrins, flavins, and melanin, generating ROS through photosensitization reactions [2,10,16,17].

### 2.2. Oxidative Stress and Reactive Oxygen Species Generation

UV-induced oxidative stress is a fundamental mechanism in the pathogenesis of photoaging [18,19,20]. Following UV exposure, a variety of ROS, including singlet oxygen (^1^O_2_), superoxide anion radical (O_2_^•−^), hydroxyl radical (^•^OH), and hydrogen peroxide (H_2_O_2_), are generated through both direct photochemical reactions and indirect photosensitization processes [21]. Both UV radiation and chronological aging contribute to ROS production; excessive ROS generation creates oxidative stress, which culminates in cellular dysfunction, lipid peroxidation, and DNA damage. High levels of ROS can initiate aging-related signaling cascades in skin cells, thereby promoting cellular senescence and, ultimately, cell death [22,23,24,25].

**Figure 1:**
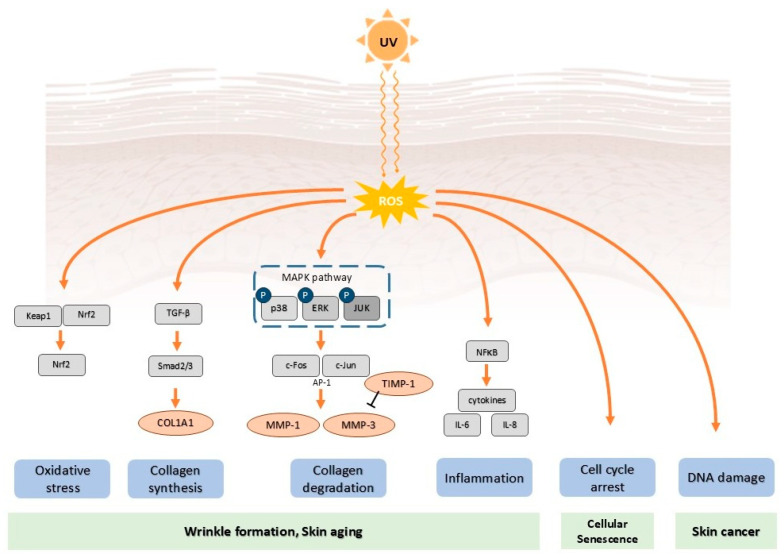
The regulation of ultraviolet (UV) exposure on skin photoaging. UV radiation generates reactive oxygen species (ROS), triggers mitogen-activated protein kinases (MAPK) activation, leading to activator protein-1 (AP-1)-mediated matrix metalloproteinase, such as matrix metalloproteinase (MMP)-1 and MMP-3 upregulation, and then causes collagen degradation. TGF-β/Smad signaling promotes collagen synthesis via COL1A1; however, UV may downregulate this pathway. UV exposure activates NF-κB, leading to the generation of inflammatory cytokines, including interleukin (IL)-6 and IL-8. It triggers nuclear factor erythroid 2-related factor 2 (Nrf2) translocation into the nucleus, providing antioxidant defense. These processes result in oxidative stress, collagen imbalance, inflammation, DNA damage, and cellular senescence, ultimately manifesting as wrinkle formation, skin aging, and even skin cancer.

This excessive ROS production leads to oxidative damage to key macromolecules such as lipids, proteins, and nucleic acids [18,26,27]. For instance, lipid peroxidation generates reactive aldehydes, including 4-hydroxynonenal and malondialdehyde, which aggravate tissue damage and inflammatory responses within the skin [28,29]. UV exposure also induces protein oxidation, resulting in the formation of protein carbonyls, advanced oxidation protein products, and cross-linked protein aggregates that impair tissue function. Furthermore, ROS-mediated DNA oxidation leads to the formation of lesions like 8-hydroxy-2′-deoxyguanosine (8-OHdG), a critical biomarker associated with mutagenesis and carcinogenesis [23,30,31,32] (Figure 1).

### 2.3. Inflammatory Responses

UV irradiation triggers a complex inflammatory cascade by activating various signaling pathways, including nuclear factor-κB (NF-κB), activator protein-1 (AP-1), and the mitogen-activated protein kinases (MAPKs), specifically p38, ERK1/2, and JNK [1,25,33]. These pathways converge to upregulate the expression of inflammatory mediators, such as interleukin-1β (IL-1β), interleukin-6 (IL-6), interleukin-8 (IL-8), tumor necrosis factor-α (TNF-α), and cyclooxygenase-2 (COX-2) [25,34] (Figure 1). The NF-κB pathway, which is activated by UV-induced oxidative stress and DNA damage, is a key regulator of these inflammatory genes. UV exposure promotes the activation of IκB kinase, which phosphorylates and subsequently degrades the inhibitory protein IκBα, permitting NF-κB to translocate to the nucleus and initiate transcription. The resulting localized inflammatory responses, mediated by immune cells and the release of pro-inflammatory cytokines, activate matrix metalloproteinases (MMPs), thereby causing the structural alterations characteristic of photoaged skin [35,36,37,38].

### 2.4. Matrix Metalloproteinases Activation and Extracellular Matrix Degradation

The degradation of the extracellular matrix (ECM) is a hallmark of photoaging, a process primarily mediated by the increased expression and activity of MMPs following the activation of the MAPK and AP-1 signaling pathways (Figure 1). MMPs, which are regulated by oxidative stress generated from sources such as UV exposure, are crucial in both intrinsic and extrinsic skin aging [1]. Key MMPs activated in photodamaged skin include collagenase (MMP-1), stromelysin (MMP-3), and gelatinase (MMP-9), which collectively degrade essential ECM components like collagen and elastin [39].

The activation mechanism is initiated when UV-induced ROS triggers the MAPK signaling cascade, which in turn activates the transcription factor AP-1. AP-1, a dimeric protein complex of the Jun and Fos families, subsequently upregulates the transcription of MMP genes, leading to accelerated collagen breakdown and wrinkle formation. Critically, AP-1 exerts a dual detrimental effect; it not only promotes ECM degradation but also suppresses new collagen synthesis by inhibiting the transforming growth factor-β (TGF-β) signaling pathway [40]. The TGF-β pathway is the major pathway regulating procollagen biosynthesis and ROS production [41]. Furthermore, excessive ROS production directly impairs fibroblast function and inhibits TGF-β signaling through the downregulation of its receptor (TβRII) and the signaling molecule Smad3, contributing to collagen loss and dermal thinning in aged skin. This cycle of repeated UV exposure and subsequent tissue injury results in the cumulative damage characteristic of photoaging [41,42,43].

### 2.5. DNA Damage

UV radiation induces various forms of DNA damage, most notably cyclobutane pyrimidine dimers (CPDs) and 6-4 photoproducts (6-4PPs) [15,31]. In response, cells activate multiple DNA repair mechanisms, including nucleotide excision repair (NER) and base excision repair (BER), to maintain genomic integrity. However, the efficiency of these repair systems progressively declines with chronological aging and can be overwhelmed by excessive UV exposure, leading to the accumulation of mutations and cellular dysfunction [44].

When DNA damage is irreparable, cells can activate cell cycle checkpoints and undergo apoptosis, often through p53-mediated signaling pathways, as a protective measure to eliminate genetically compromised cells [45,46]. Nevertheless, chronic UV exposure can subvert this surveillance system, allowing cells with unrepaired DNA damage to survive. The persistence of these genetically compromised cells increases the risk of malignant transformation and is a foundational step in the development of skin cancer [47,48].

### 2.6. Cellular Senescence

Cellular stress induced by UV radiation, including persistent DNA damage, telomere dysfunction, and oxidative stress, can trigger a state of irreversible growth arrest known as cellular senescence [49,50,51]. Senescent cells accumulate in photoaged skin tissues and acquire a distinct senescence-associated secretory phenotype (SASP). The SASP is characterized by the secretion of a wide array of pro-inflammatory cytokines, chemokines, growth factors, and matrix-degrading enzymes [52,53]. The secretion of cytokines contributes to chronic inflammation and tissue dysfunction, triggers the aging process, and promotes further cellular damage and deterioration. It creates a chronic, low-grade inflammatory microenvironment that degrades the surrounding ECM, disrupts normal tissue homeostasis, and can even induce senescence in neighboring cells, thereby amplifying and perpetuating the aging process [51,54].

### 2.7. Advanced Glycation End Products and Skin Aging

Glycation, a non-enzymatic reaction between reducing sugars and proteins or lipids, is a contributing factor to both intrinsic and extrinsic skin aging [55,56,57]. Over time, this process leads to the formation and accumulation of advanced glycation end products (AGEs). Within the skin, AGEs irreversibly cross-link long-lived proteins like collagen and elastin, resulting in increased stiffness and reduced elasticity of the dermal matrix [58,59].

The human body produces glycation, which affects collagen and elastin molecules. Receptor for advanced glycation end products (RAGE) modulates the intracellular signaling pathway by regulating cell signals to generate cytokines and amplifying oxidative stress to activate the NF-κB mediated by the MAP kinase pathway [59,60,61,62]. The binding of AGEs to RAGE activates intracellular signaling cascades, including the MAPK pathway, which in turn stimulates the NF-κB transcription factor. Activated NF-κB then drives the production of pro-inflammatory cytokines, promoting a state of chronic inflammation that accelerates skin aging and damage [63,64]. Importantly, exposure to UV radiation has been shown to accelerate the deposition of AGEs in the skin, creating a vicious cycle where photoaging and glycation potentiate [65].

## 3. Djulis and Phytochemical Composition of Djulis Extract

Djulis (*Chenopodium formosanum*), also known as Taiwanese quinoa, is a pseudocereal native to Taiwan, traditionally cultivated by Taiwanese aborigines for more than 100 years. This ruby-colored grain belongs to the Amaranthaceae family [66,67]. Djulis is consumed by indigenous communities and is a Taiwanese endemic “superfood” with functional properties. At the same time, it is recognized for its nutritional value—rich in protein, dietary fiber, calcium, and essential amino acids like lysine. Djulis is rich in phenolic compounds and phytoecdysteroids, and has been reported to have anti-diabetic, anticancer, anti-aging, anti-inflammation, and hepatoprotective properties. The therapeutic potential of djulis in dermatology stems primarily from a diverse array of bioactive phytochemicals [9]. The composition and concentration of these phytochemicals can vary significantly depending on the plant’s maturity, color, and the specific tissue type (e.g., leaf or seed) [68,69].

The primary bioactive constituents of djulis relevant to its anti-photoaging effects are listed as follows.

### 3.1. Phenolic Compounds

Djulis is a rich source of phenolic acids and flavonoids [68]. Major phenolic acids identified include chlorogenic acid, gallic acid, ferulic acid, and vanillic acid [70]. Key flavonoids include rutin and epicatechin. These compounds are largely responsible for the extract’s potent antioxidant activity. Polyphenols are well-known antioxidants and skin protectors by possessing strong free radical scavenging activity [71,72], reducing inflammation, and absorbing UV radiation to provide skin photoprotection [73,74]. Rutin is a flavonoid widely distributed in fruits and vegetables and the representative polyphenol in djulis indicated by previous studies [75,76]; several reports have demonstrated the biological effects of rutin on ROS-induced skin aging [77] and suggested that rutin effectively inhibits the formation of AGEs on collagen synthesis [78].

### 3.2. Betalains

The characteristic red color of djulis grains comes from betalains, with betanin being the principal pigment. Betalains are powerful antioxidants known to mitigate inflammatory diseases, protect against oxidative damage, and inhibit the oxidation of lipids [67]. It has been reported that betalains can reduce the sensitivity of inflammatory diseases [79], protect the liver from damage [80], and inhibit melanoma cell proliferation [81]. In addition, betalains have also been found to inhibit linoleate peroxidation induced by cytochrome C and H_2_O_2_-activated low-density lipoprotein oxidation [82].

### 3.3. Phytoecdysteroids

Djulis contains phytoecdysteroids, a class of plant-derived steroids with notable bioactivity [75,76,83,84]. The most abundant of these is 20-hydroxyecdysone, which has been reported to inhibit MMP activity, reduce intracellular ROS production, and decrease collagenase activity, making it highly relevant for combating photoaging [85]. In addition, 20-hydroxyecdysone isolated from *Chenopodium quinoa* seeds possesses strong inhibition activity against collagenase and the DPPH free radicals, and a potent ability to chelate iron ions [86].

These phytochemicals endow djulis extract with a range of biological activities, including antioxidant, anti-aging, hepatoprotective, anti-obesity, and anticarcinogenic effects. The subsequent sections will detail the mechanisms through which these activities counteract the processes of skin photoaging.

## 4. Bioactivity of Djulis Extract

**Figure 2:**
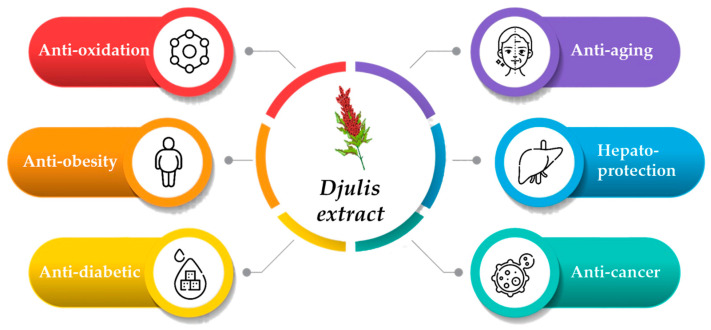
The biological activities of djulis extracts. Djulis displays antioxidant, anti-aging, hepatoprotective, anti-obesity, anti-diabetic, and anticarcinogenic effects [58,67,70,75,76,84,87,88,89,90,91,92].

Djulis has attracted increasing attention based on its antioxidant properties [67,87,88]. Previous studies have reported that djulis displays several biological activities, including anti-aging [58,70,89], hepatoprotective [87,88,90,91], anti-obesity and anti-diabetic [75,76,92], and even anticarcinogenic effects [84] (Figure 2). The following is the bioactivities and mechanisms of action of djulis (Table 1).

### 4.1. Antioxidant Activity

Djulis extract exhibits potent and multifaceted antioxidant activity, which is foundational to its anti-photoaging effects [68]. Its efficacy has been demonstrated through various in vitro assays, revealing multiple underlying mechanisms, including hydrogen atom transfer, single electron transfer, and metal chelation [93,94]. Unhulled djulis extract, in particular, shows significant scavenging capabilities against physiologically relevant ROS such as superoxide anion, hydrogen peroxide, and hydroxyl radicals at concentrations of 100–1000 μg/mL [58].

A key aspect of its antioxidant profile is its strong ferrous ion chelating activity. By sequestering ferrous ions (Fe^2+^), the extract can effectively inhibit the Fenton reaction, a major source of highly damaging hydroxyl radicals in cells. This metal-chelating property contributes significantly to its overall protective efficacy [68]. While the hull of djulis contains numerous active components, studies have shown that unhulled djulis extract possesses higher total phenolic and flavonoid content, correlating with its robust antioxidant capacity. Critically, this chemical antioxidant activity translates to a direct biological benefit: djulis extract at concentrations above 200 μg/mL significantly reduces the formation of intracellular ROS in human dermal fibroblasts (Hs68) and keratinocytes (HaCaT) following UVB irradiation [58].

### 4.2. Activation of the Antioxidant Defense System Nrf2/HO-1 Signaling Pathway

In addition to its free radical-scavenging activity, 100–250 μg/mL djulis extract reinforces the skin’s endogenous antioxidant defenses by modulating the nuclear factor erythroid 2-related factor 2 (Nrf2) signaling pathway [58]. Nrf2 is a master transcription factor that regulates the expression of a suite of antioxidant and cytoprotective genes, playing a crucial role in cellular defense against UV-mediated photodamage [95].

Upon exposure to cellular stress, phytochemicals in the extract facilitate the translocation of Nrf2 from the cytoplasm into the nucleus [22,96]. Once in the nucleus, Nrf2 activates the transcription of Phase II detoxifying enzymes and antioxidant proteins, including the highly protective enzyme heme oxygenase-1 (HO-1). Studies have confirmed that djulis extract treatment significantly increases the protein expression of HO-1 in skin cells. By upregulating the Nrf2/HO-1 axis, djulis extract enhances the cell’s intrinsic capacity to neutralize oxidative stress and prevent downstream damage, providing a sustained protective effect [58].

### 4.3. Regulation of MAPK/Matrix Metalloproteinases/Collagen Pathway

UV-induced excess ROS activates MAPKs, results in the transcriptional regulation of MMPs, and culminates in the degradation of collagen and elastin, subsequently leading to photoaging. UV radiation activates MAPK pathways (ERK1/2, p38, and JNK), leading to phosphorylation and activation of Jun and Fos family proteins that comprise the AP-1 transcription factor complex [1,3,97,98]. Djulis extract inhibits MAPK activation and subsequent AP-1-mediated gene transcription, reducing expression of inflammatory mediators and matrix metalloproteinases [70].

Djulis extract at 150 μg/mL was investigated for the inhibition of MMPs protein expression and activity to present the anti-photoaging activity [58,70]. Djulis extract at a concentration range of 50–250 μg/mL demonstrates potent inhibitory effects against matrix metalloproteinase expression and activity, to prevent ECM degradation after UV exposure [58,70]. The extract acts on the MMP regulation pathway, including transcriptional control, post-translational modification, and direct enzyme inhibition to ameliorate UV-induced skin damage.

Djulis extract (150 μg/mL) treatment significantly reduces MMP-1 production in UVB-irradiated HaCaT cells, with dose-dependent inhibition [70]. In addition, 100–250 μg/mL djulis extract also inhibited MMP-1, 3, and 9 expression and induced TIMP-1 expression in Hs68 after UVB exposure [58]. This matrix protective effect helps preserve collagen integrity and prevents the structural alterations characteristic of photoaged skin. Djulis extract inhibits AP-1-mediated MMP-1 gene expression through suppression of MAPK signaling pathways. The AP-1 binding site in the MMP-1 promoter represents a critical regulatory element for UV-induced collagenase expression, and its inhibition by djulis extract effectively prevents collagen degradation [58].

Post-translational mechanisms involve modulation of MMP activation processes, and MMPs are secreted as inactive zymogens that require proteolytic activation for catalytic activity [99]. Djulis extract interferes with this activation cascade, preventing conversion of pro-MMPs to their active forms and thereby limiting ECM degradation capacity [58]. Direct enzyme inhibition represents an additional mechanism whereby phenolic compounds in djulis extract directly interact with MMP active sites, providing competitive or non-competitive inhibition. In addition, the metal-chelating properties of phenolic compounds may contribute to this effect by sequestering zinc ions essential for MMP catalytic activity. The TGF-β/Smads pathway is the major pathway regulating procollagen biosynthesis and ROS production [41]. Oxidative stress inhibits TGF-β signaling by downregulating Smad3, which contributes to the loss of collagen content in aged skin [40]. Djulis extract at 200–250 μg/mL increased TGF-β and Smad3 expression to enhance the total collagen content [58].

The accumulation of UV and AGEs stress causes collagen degradation and interferes with each other during intrinsic and extrinsic aging [55,100]. The downregulation of MMPs protein expression by djulis extract inhibited collagen degradation [58]. Djulis extract maintains collagen content and enhances collagen gene expression in UV radiation- and glycation-stressed fibroblasts [58]. Animal studies confirm these protective effects, with both oral and topical djulis extract (40 and 100 mg/kg body weight) applications helping preserve skin structure and reduce UV-induced epidermal thickening [70].

### 4.4. Anti-Inflammatory Activity

Djulis extract exhibits potent anti-inflammatory properties, effectively mitigating the inflammatory cascade triggered by UV radiation [58,70]. In vitro studies using UVB-irradiated keratinocytes have shown that the extract (100–150 μg/mL) significantly reduces the production of key pro-inflammatory mediators, such as IL-6, in a dose-dependent manner [70].

The primary anti-inflammatory mechanism involves the inhibition of the NF-κB and MAPK signaling pathways, which are critical drivers of UV-induced inflammation [25,101]. The extract’s phenolic constituents, particularly rutin and chlorogenic acid, are believed to be major contributors to this effect. These compounds can inhibit the activation of IκB kinase (IKK), thereby preventing the degradation of IκBα and blocking the nuclear translocation of NF-κB. By suppressing the inflammatory pathways, djulis extract at concentrations of 100–150 μg/mL reduces the expression of inflammatory genes and ameliorates the skin’s response to UV damage [70].

### 4.5. Cellular Protection

Through its combined antioxidant, anti-inflammatory, and matrix-preserving activities, djulis extract at the concentration range of 50–250 μg/mL provides comprehensive cellular protection against UV-induced damage [58,70]. Treatment with the extract significantly enhances the viability of UVB-irradiated keratinocytes. It protects cells from apoptosis, as evidenced by a reduction in the sub-G1 cell population, which is indicative of apoptotic cells. This cytoprotective effect is achieved by stabilizing mitochondrial function, modulating apoptotic pathways, and enhancing the overall cellular defense system. Importantly, this protection does not appear to compromise genomic safety, as the extract does not alter 5-methylcytosine levels, suggesting it does not promote the survival of cells with potentially malignant DNA damage [70].

### 4.6. Inhibition of Advanced Glycation End Products Formation

Beyond its direct photoprotective effects, djulis extract also combats skin aging by targeting glycation-induced damage, a process exacerbated by UV exposure [58]. The extract demonstrates significant anti-glycation properties, protecting human skin fibroblasts from damage induced by AGEs like Nε-carboxymethyl-lysine (CML). Its mechanism involves breaking the vicious cycle where oxidative stress and glycation reinforce each other. First, by reducing UV-induced ROS, the extract simultaneously inhibits a key accelerator of AGE formation. Second, the extract directly interferes with AGE-mediated signaling. It significantly downregulates the protein expression of the AGE receptor (RAGE) in AGE-treated fibroblasts [58]. By reducing RAGE levels, the extract dampens the downstream inflammatory and oxidative signaling, further protecting cells from glycation-induced stress. This dual action against both UV- and glycation-induced damage highlights a novel and critical mechanism for its comprehensive anti-aging potential [102].

### 4.7. Clinical Trial Design and Validation Studies

Long-term safety studies are critical given the intended chronic use of photoprotective agents. Candidate biomarkers include AGE levels measured by skin autofluorescence, inflammatory mediators in skin biopsies, and oxidative stress markers in blood or urine samples [102]. Consuming djulis products (containing 2% djulis extract) for 4 or 8 weeks may increase skin moisture, brightness, elasticity, and collagen content, while decreasing wrinkles and spots [103,104]. Djulis extract drink enhanced skin hydration (17.8%), brightness (5.4%), collagen content (22.3%), and improved crow’s feet (14.9%), texture (9.9%), wrinkles (29.3%), pores (10.4%), spots (9.9%) after 8 weeks [103]. In addition, consumption of the djulis drinks significantly increased the levels of superoxide dismutase (9.5%) and catalase (124.8%) in serum after 8 weeks [105]. Topical application of 0.0625% to 0.25% djulis leaf extract for 20 min may increase skin moisture levels and firmness, improve skin texture, and reduce oil secretion [68]. The effects of djulis products on skin were shown in Table 2.

## 5. Safety of Djulis

The safety profile of djulis extract has been rigorously evaluated through an extensive series of in vitro and in vivo studies, alongside its long history of traditional use.

### 5.1. In Vitro Studies

In human skin fibroblasts (Hs68 cells), djulis extract demonstrated excellent biocompatibility across a concentration range of 50–250 μg/mL, exhibiting no cytotoxic effects even after 24-h exposure [58,70]. These findings were corroborated in CCD-966SK fibroblasts, where cell viability remained unaffected at concentrations ranging from 0.125% to 1% [89]. Furthermore, djulis extract up to 150 μg/mL did not induce cell death in human keratinocytes (HaCaT cells) [58]. DNA methylation analysis provided crucial insights, revealing that the percentage of 5-methylcytosine remained stable after djulis treatment [70]. This indicates that djulis extract does not alter DNA integrity or promote the survival of precancerous DNA-damaged cells, a significant finding for its potential applications [70].

### 5.2. Animal Studies

In animal models, oral administration of djulis extract to female BALB/c mice at doses ranging from 40–100 mg/kg body weight for 14 consecutive days resulted in no observable adverse reactions or toxicity manifestations [66]. Animals consistently maintained normal behavior, body weight progression, and physiological parameters, underscoring the excellent oral tolerability of djulis extract [66]. Additionally, topical application of 8 mg djulis extract (approximately 200 μL of emulsion) for seven consecutive days demonstrated excellent skin tolerance, with no signs of irritation, inflammation, or adverse dermal reactions [70].

### 5.3. Traditional Use and Clinical Trial

Further support for the safety of djulis is derived from its extensive traditional use among Taiwanese indigenous populations, who have consumed this grain as a staple food for over 100 years. It is commonly cooked with rice or millet and has been incorporated into various food products, including bakery items, noodles, and functional beverages, without any reported safety concerns [66,67,68].

Collectively, these evidence-based safety parameters establish clear guidelines for the use of djulis extract. For topical applications, concentrations ranging from 50–250 μg/mL have been confirmed as safe and effective [105]. For oral supplementation, doses of 40–100 mg/kg body weight provide therapeutic benefits without adverse effects [89].

This comprehensive safety assessment provides a robust scientific foundation for the development of djulis extract as a functional food ingredient, nutritional supplement, or cosmetic component in various health and wellness applications.

## 6. Conclusions

Botanical extracts have gradually become a trend in recent years because more people are beginning to recognize the concept of nature and sustainability. Additionally, the primary advantage of botanical extracts is their complex composition and the synergistic effect of related compounds with multiple activities to obtain greater efficacy. This review establishes djulis (*Chenopodium formosanum*) extract as a multifunctional natural agent with potential for photoaging prevention and treatment. The accumulated evidence from in vitro, ex vivo, and in vivo studies demonstrates that djulis extract provides comprehensive protection against UV-induced skin damage through antioxidant activity. The extract provides comprehensive UV protection through sophisticated cellular defense activation, enhancing Nrf2/HO-1 antioxidant pathways while delivering potent free radical scavenging activity (IC_50_ = 84.7 ± 13.0 μg/mL). In addition, djulis also exhibited anti-glycation properties that prevent AGE formation and RAGE-mediated skin tissue damage. Djulis contains betalains (betanin 14.5 ± 0.4 mg/g) and phenolic compounds (rutin 19.5 ± 0.6 mg/g), enabling multi-target therapeutic effects within optimal safety windows (50–250 μg/mL topical, 40–100 mg/kg body weight oral). Djulis extract represents a multifunctional agent with scientifically-proven mechanisms and a safety profile, which may be used for anti-aging and anti-photoaging in functional foods and cosmeceutical applications.

## Figures and Tables

**Table 1:** Biological activity of djulis (*Chenopodium formosanum*) and derived products in vitro and in vivo studies.

Plant	Extract Solvent	Cell Model	Animal Model	Anti-Aging Biological Activity	Reference
Djulis seeds	Water	Inducer: UVB Cell line: HaCaT cells (immortalized human keratinocyte cell line)	Inducer: UVB Animal: BALB/c mice	Alleviate cell deaths and decrease ROS levels Reverse IL-6 and MMP-1 production in cells Improve epidermis thickness in mice	[70]
Unhulled djulis	Water	Cell line: CCD-966SK cells (human skin fibroblast cell line)	-	Increase wound healing ability Promote collagen secretion Decrease AGEs formation Regulate skin-barrier-related genes (TGM1, KRT1, KRT10), antioxidant-related genes (SOD2), collagen-related genes (COL1A2, MMP-9) expression	[89]
Unhulled djulis	Water	Inducer: UVB and CML Cell line: Hs68 cells (human skin fibroblast cell line)	-	Strong ROS and free radical scavenging effects Initiate Nrf2/HO-1 antioxidant defense system Modulate MAPK and TGF-β signaling pathway Attenuated glycation stress Promote collagen synthesis and improve collagen degradation	[58]
**Plant**	**Extract Solvent**	**Cell Model**	**Animal Model**	**Hepatoprotective Biological Activity**	**Reference**
Djulis	Water	Inducer: t-BHP Cell line: HepG2 cells (human hepatoma cell line)	-	Decrease lactate dehydrogenase (LDH) release Decrease ROS levels and lipid peroxidation Activate glutathione activity Inhibit IκB-α degradation Regulating Bcl-2/Bax, PARP, and caspase-3 cell apoptosis pathway Enhance mitochondrial membrane potential	[87]
Djulis	Water	-	Inducer: CCl_4_ Animal: Wistar rats	Decrease lipid peroxidation Activate superoxide dismutase (SOD) and glutathione (GSH) activities, enhance cytochrome P450 2E1 (CYP2E1) concentrations Attenuate DNA damage	[90]
Djulis	Water	-	Inducer: EtOH Animal: Wistar rats	Decrease lipid peroxidation Activate catalase and GSH activities Enhance CYP2E1 activity Attenuate DNA damage	[91]
Djulis bran	Water or ethanol	-	Inducer: CCl_4_ Animal: BALB/c mice	Decrease ROS levels and lipid peroxidation Activate SOD, CAT, and GSH activities Inhibit TNF-α, IL-6, and TGF-1 levels	[88]
**Plant**	**Extract Solvent**	**Cell Model**	**Animal Model**	**Anti-Obesity and Anti-Diabetic Biological Activity**	**Reference**
Djulis	Ethanol	-	Inducer: High-fat diet Animal: C57BL/6 mice	Alleviate hyperlipidemia - decrease plasma cholesterol levels - decrease triglyceride levels Alleviate hyperglycemia - improve glucose tolerance - ameliorate insulin response	[92]
Djulis	Water	Cell line: SVEC endothelial cells	Animal: Spontaneously hypertensive rats and Wistar Kyoto rats	Prevent hypertension - Decrease ROS levels - Increase nitric oxide (NO) production - Activate endothelial nitric oxide (eNOS) synthase expression - Promote COX-2 expression - Increase prostacyclin (PGI_2_) formation - Decrease peroxynitrite (ONOO^−^) production - Inhibit angiotensin converting enzyme activity	[76]
Djulis	Ethanol	Cell line: 3T3-L1 cells (dipocyte cell line)	-	Prevent adipogenesis - Inhibit lipid accumulation - Decrease triglyceride levels - Decrease glycerol-3-phosphate dehydrogenase (GPDH) activity - Decrease PPAR, C/EBP and SREBP-1c gene expression	[75]
**Plant**	**Extract Solvent**	**Cell Model**	**Animal Model**	**Anticarcinogenic Biological Activity**	**Reference**
Djulis husk	Ethanol	Cell line: HepG2 cells (human hepatoma cell line)		Increase LDH release Increase ROS generation Attenuate mitochondrial transmembrane potentials Regulating Bcl-2/Bax, PARP, and caspase-3 cell apoptosis pathway Slow down the cell cycle at the Sub-G0 phase	[84]

**Table 2:** Clinical trials of the effect of djulis (*Chenopodium formosanum*) products on skin parameters.

Participants and Groups	Treatment	Duration	Skin Parameters	Reference
50 subjects (average age 53 years) (25 subjects in collagen drink group) (25 subjects in placebo drink group)	Collagen drink (main ingredient: 12% fish collagen, 2% djulis extract, 1% green caviar) Placebo drink	28 days	Upregulation by collagen drink: Skin moisture (+27.54%) Skin elasticity (+11.14%) Skin gloss (+26.14%) Downregulation by collagen drink: Skin spot (−12.42%) Skin wrinkle (−19.92%) Skin roughness (−30.79%) Skin erythema (−23.4%)	[104]
50 subjects (35–50 years old) (25 subjects in collagen drink group) (25 subjects in placebo drink group)	Collagen drink (main ingredient: 11% fish collagen, 2% djulis extract) Placebo drink	8 weeks	Upregulation by collagen drink: Skin hydration (+17.8%) Skin brightness (+5.4%) Skin collagen content (+22.3%) Downregulation by collagen drink: Crow’s feet (−14.9%) Skin texture (−9.9%) Skin wrinkle (−29.3%) Skin pore (−10.4%) Skin spot (−9.9%)	[103]
30 subjects (35–55 years old) (15 subjects in djulis functional drink group) (15 subjects in placebo drink group)	Djulis functional drink Placebo drink	8 weeks	Upregulation by djulis functional drink: Skin moisture (+13.3%) Skin brightness (+3.8%) Skin elasticity (+13.2%) Skin collagen content (+33.7%) Downregulation by djulis functional drink: Crow’s feet (−21.8%) Skin texture (−12.1%) Skin wrinkle (−11.0%) Skin pore (−1.4%)	[105]
30 subjects	Gel formula with djulis leaf extract (0.0625% to 0.25%)	20 min	Increase skin hydration, elasticity, radiance, firmness Decrease skin oiliness, texture irregularities, discoloration	[68]

## Data Availability

No new data were created or analyzed in this study. Data sharing is not applicable to this article.

## Abbreviations

The following abbreviations are used in this manuscript:

6-4PPs	6-4 photoproducts
8-OHdG	8-hydroxy-2′-deoxyguanosine
AGEs	advanced glycation end products
AP-1	activator protein-1
CML	Nε-carboxymethyl-lysine
COX-2	cyclooxygenase-2
CPDs	cyclobutane pyrimidine dimers
ECM	extracellular matrix
IKK	IκB kinase
IL	interleukin
MAPKs	mitogen-activated protein kinases
MMPs	matrix metalloproteinases
NF-κB	nuclear factor-κB
RAGE	receptor of advanced glycation end products
ROS	reactive oxygen species
SASP	senescence-associated secretory phenotype
TNF-α	tumor necrosis factor-α
UV	ultraviolet
